# Recent Advances in Thyroid Hormone Regulation: Toward a New Paradigm for Optimal Diagnosis and Treatment

**DOI:** 10.3389/fendo.2017.00364

**Published:** 2017-12-22

**Authors:** Rudolf Hoermann, John E. M. Midgley, Rolf Larisch, Johannes W. Dietrich

**Affiliations:** ^1^Department for Nuclear Medicine, Klinikum Lüdenscheid, Lüdenscheid, Germany; ^2^North Lakes Clinical, Ilkley, United Kingdom; ^3^Medical Department I, Endocrinology and Diabetology, Bergmannsheil University Hospitals, Ruhr University of Bochum, Bochum, Germany; ^4^Ruhr Center for Rare Diseases (CeSER), Ruhr University of Bochum, Bochum, Germany; ^5^Ruhr Center for Rare Diseases (CeSER), Witten/Herdecke University, Bochum, Germany

**Keywords:** setpoint, thyroid homeostasis, thyroid-stimulating hormone, levothyroxine treatment

## Abstract

In thyroid health, the pituitary hormone thyroid-stimulating hormone (TSH) raises glandular thyroid hormone production to a physiological level and enhances formation and conversion of T4 to the biologically more active T3. Overstimulation is limited by negative feedback control. In equilibrium defining the euthyroid state, the relationship between TSH and FT4 expresses clusters of genetically determined, interlocked TSH–FT4 pairs, which invalidates their statistical correlation within the euthyroid range. Appropriate reactions to internal or external challenges are defined by unique solutions and homeostatic equilibria. Permissible variations in an individual are much more closely constrained than over a population. Current diagnostic definitions of subclinical thyroid dysfunction are laboratory based, and do not concur with treatment recommendations. An appropriate TSH level is a homeostatic concept that cannot be reduced to a fixed range consideration. The control mode may shift from feedback to tracking where TSH becomes positively, rather than inversely related with FT4. This is obvious in pituitary disease and severe non-thyroid illness, but extends to other prevalent conditions including aging, obesity, and levothyroxine (LT4) treatment. Treatment targets must both be individualized and respect altered equilibria on LT4. To avoid amalgamation bias, clinically meaningful stratification is required in epidemiological studies. In conclusion, pituitary TSH cannot be readily interpreted as a sensitive mirror image of thyroid function because the negative TSH–FT4 correlation is frequently broken, even inverted, by common conditions. The interrelationships between TSH and thyroid hormones and the interlocking elements of the control system are individual, dynamic, and adaptive. This demands a paradigm shift of its diagnostic use.

## Introduction

In the healthy body, multiple interlocking biochemical mechanisms interact homeostatically both to achieve biological equilibrium and to respond suitably to any challenges, internal or external, that might occur. However, the way in which different individuals maintain a steady homeostatic state varies considerably, including their appropriate reaction to any changes that may be demanded of the system. In this regard, the possible biochemical expressions of a healthy framework within which viable variations of response can occur have finite limits (1). Appropriate reactions in individuals are defined by their own unique solutions both to homeostatic equilibrium and to biochemical challenges, which are much more closely constrained than those permissible over the whole population. This has profound implications as to how disequilibria are confronted by individual responses to the challenge or therapeutic interventions administered to obvert it.

The classical definitions of hypothyroidism and hyperthyroidism reflect this concept when referring to inadequate—either reduced or exaggerated—supply and response to thyroid hormones (2). Pathophysiological conditions or diseases may arise at various levels including deficiencies of hormone supply, alterations of control or resistance to cellular responses to the hormones (3–5). Thyroid gland failure represents a more restricted view, termed primary hypothyroidism. Current diagnostic definitions of thyroid disease are primarily laboratory based, and include subclinical dysfunctions that are dissociated from treatment recommendations (6–9). In this review, we revisit underlying principles of thyroid control, aligning or contrasting them with the current use of thyroid-stimulating hormone (TSH) in the diagnosis and treatment of thyroid diseases.

## The Current Paradigm of Thyroid Diagnosis

Two events have shaped the current paradigm of thyroid function testing (1) the discovery of negative feedback by thyroid hormones on pituitary TSH and (2) methodological advances in sensitively measuring TSH (6, 10, 11). If TSH serum concentrations provided a more sensitive and accurate mirror image of thyroid hormone status than thyroid hormones themselves, this would be an ideal diagnostic tool. This argument emerged in the 1980s, and was readily accepted by clinicians (12, 13). TSH measurement subsequently gained a dominant role in thyroid function testing, facilitating cost effective disease screening, introducing new definitions of subclinical hypothyroidism or hyperthyroidism, and delivering biochemical treatment targets (14–17). A plethora of epidemiological studies then associated TSH concentrations with clinical outcomes (18–21). This resulted in a questionable paradigm elevating an indirect controlling parameter to such prominence and dominance as expressed in current guidelines for diagnosis and treatment of thyroid diseases (14–16). With the holy grail in the hand of scientists, clinically orientated approaches contradicting the TSH paradigm and noting discrepancies between a biochemically euthyroid and clinically hypothyroid status were readily dismissed (22–26). However, concerns have resurfaced in the light of recent basic and clinical studies revealing fundamental issues with the interpretation of TSH measurements and documenting unsatisfactory improvement in the quality of life of patients treated for hypothyroidism according to the current TSH paradigm (1, 27–29).

## Shifting the Paradigm

Is the TSH concentration at all times a reliable mirror image of the thyroid hormone status of the patient?

**Figure 1:**
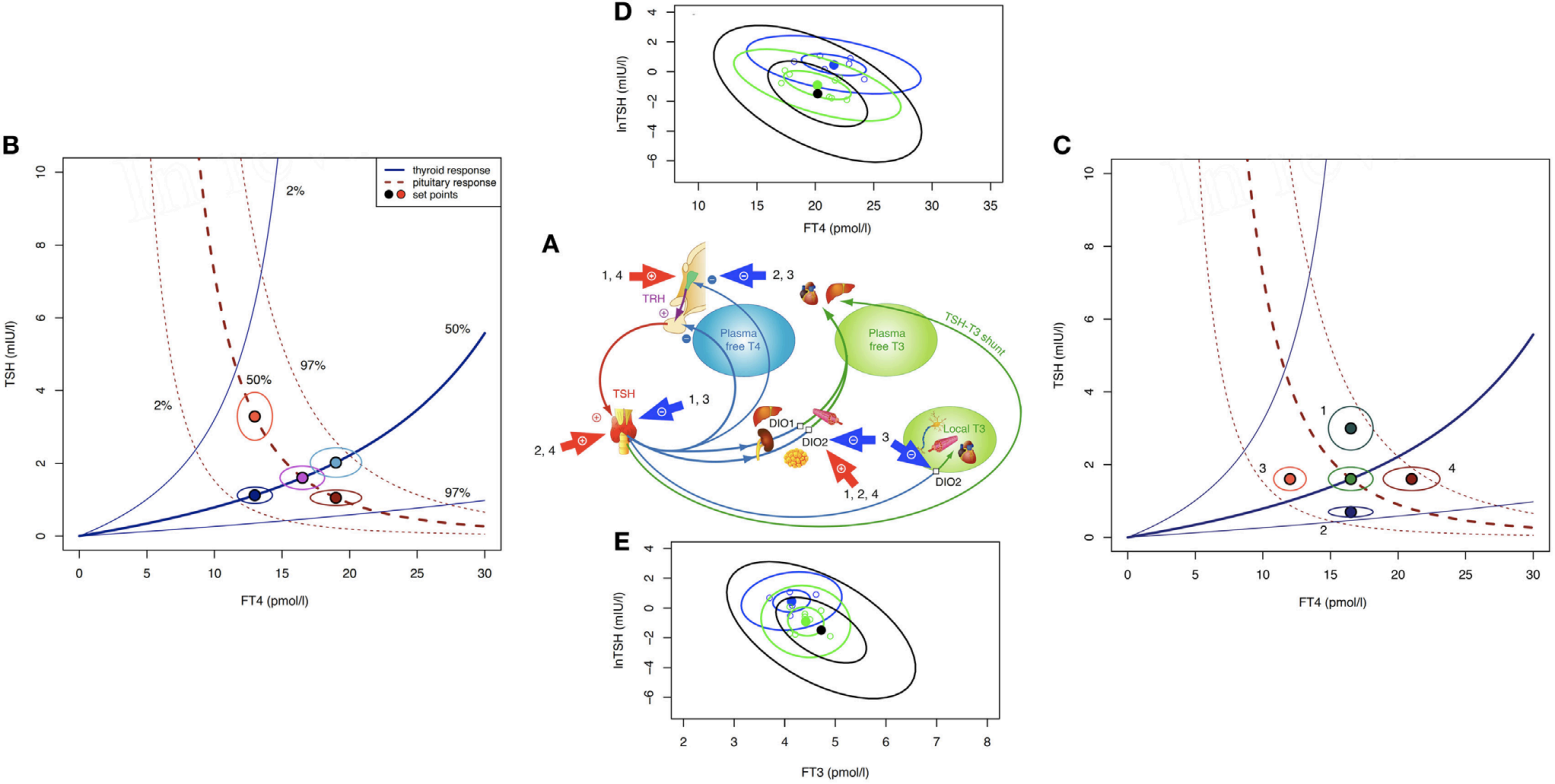
Theoretical and observed relationships between thyroid-stimulating hormone (TSH) and thyroid hormones. The central system provides an integrated solution to the location of the setpoint (settling point) of thyroid homeostasis, as determined by the various genetic, epigenetic, and allostatic parameters (27, 36). This includes monogenic factors such as polymorphic variants of receptors and transporters, changes in deiodinase activity or variations in T4 production efficiency, and environmental impacts, e.g., nutritional factors, body weight, body composition, age, and extends to diseases of the thyroid and other organs where severe disequilibria may arise and a “euthyroid” solution is not achieved (27, 36). **(A)** Schematic overview of main regulatory pathways and control loops in the hypothalamic–pituitary–thyroid regulation. External factors of influence include (1) obesity (hyperthyrotropinemia), (2) pregnancy (hCG-mediated suppression of TSH release and stimulation of T4 secretion), (3) non-thyroidal illness (NTI) (both pituitary and thyroid function down-regulated), and (4) certain psychiatric diseases (both pituitary and thyroid function stimulated). **(B)** The response of the thyroid to TSH by hormone release and corresponding feedback on pituitary TSH secretion produces a finite equilibrium solution, thereby defining interlocking TSH–FT4 pairs (setpoints). These are characteristic for each person and show only little variation unless disturbed by internal or external strain. In the event of progressive thyroid capacity stress, irrespective and independent of other influences, setpoints for FT4 and TSH translocate along a homeostatic pituitary response curve (isocline) unique for the individual. Hence, in the case of a diminished or exaggerated pituitary response the translocation moves along the thyroid isocline. The open circles indicate the expected variation (10% for FT4 and 30% for TSH) surrounding the individual setpoint. The percentiles for the isoclines of the response curves were derived from previous data in a healthy sample and the included area between the 2.5 and 97.5 percentiles represents the population’s reference range (37). **(C)** Influences additional to direct thyroid activity change (e.g., allostatic changes such as obesity, age, pregnancy, and NTI) may have dislocating effects on isoclines and setpoints. **(D)** Observed TSH–FT4 pairs (setpoints) in two individual patients (blue and green symbols) and the averaged group of 250 patients (black ellipse) followed long term on stable treatment conditions. Data ellipses indicate the 50 and 95% confidence limits for the setpoint. Levothyroxine dose was 100 μg/day, 1.5 µg/kg body weight for each of the individual patients, and 1.5 (SD 0.27) μg/kg for the whole group. Data are from a published longitudinal study (38). **(E)** Observed TSH–FT3 pairs depicted in the same patients as in **(D)**. FT3 concentrations vary with conversion efficiency and impact on the location of the TSH–FT4 setpoint (38).

Firstly, we must note that this question reverses causality of process. While TSH is subject to negative feedback by thyroid hormones predominantly FT4, thereby reflecting on the thyroid hormone status, its primary physiological role is to elevate the hypothyroid to the euthyroid state (1). Without appropriate stimulation by TSH, the basal hormone output by the thyroid gland remains insufficient, as becomes apparent in pituitary deficiency (secondary hypothyroidism) (4). Conversely, in thyroid health TSH raises the glandular hormone production to its physiological level, and it also enhances the formation of the biologically more active T3 and its conversion from T4 (30–32). The integration of the TSH–T3 shunt into the homeostatic control of the thyroid–pituitary axis is illustrated in Figure 1A. Feedback details have been discussed elsewhere (27). Clinical studies and theoretical modeling suggest that the shunt facilitates FT3 stability against variations in the glandular T4 output, and its absence may lead to T3 production instability in athyreotic patients (31, 33). This relational control loop operates still within the euthyroid TSH range (34). It also appears to mediate subtle expressions of central control over peripheral tissues, such as circadian or seasonal variation in FT3, but not FT4, the former lagging the TSH rhythm (35). In thyroid autoimmune disease, the feedforward control increases T4 to T3 conversion, compensating for latent thyroid failure until the disease has progressed beyond tolerance (34).

Pathophysiological challenges other than thyroid disease may disturb the equilibria between TSH and thyroid hormones, requiring setpoint adjustment of the hypothalamic–pituitary–thyroid axis (Figures 1A–C). Weight gain or loss, alterations in body composition, and aging may cause profound changes (Figure 1C), frequently switching the control mode from negative feedback to tracking FT4 rather than opposing it (36, 39, 40). This inversion of the TSH–FT4 correlation is clinically important to recognize, but it may be missed or misinterpreted as subclinical hypothyroidism when relying on TSH measurement as sole diagnostic test. Several mechanisms provide a physiological interface for shifting the control modes of the hypothalamic–pituitary–thyroid axis (36, 39–46). While detailed examination of the complex subject is beyond the scope of this article and dedicated reviews are available (36, 39, 41, 43, 45, 46), a brief explanation may be helpful. Hypothalamic tancytes strongly express the relatively more T3- than T4-affine thyroid hormone transporter MCT8 and deiodinase type 2 and 3, enabling them to sense FT3 and FT4 levels (41, 44–46). They also respond to alterations in the energetic and metabolic needs of the body and interact with TRH neurons (41, 42, 44–48). Fat cells release adipokines such as leptin into the circulation which directly or indirectly stimulate pituitary TSH secretion (36, 39, 40, 42, 45). In a vicious cycle, TSH may promote leptin release through TSH receptor activation on adipocytes (49). The controlling system stays informed on fat and energy reserves, and may act on adjusting energy expenditure accordingly. While rising with weight gain TSH decreases again after weight loss (36, 39, 50, 51). Central and peripheral deiodinases are also sensitive to nutritional factors and body composition, adjusting T4 to T3 conversion to maintain T3 stability under varying conditions (39, 40). As transporters, deiodinases and thyroid hormone receptors subtypes differ in both their expressions and T3 affinities, central responses may readily diverge from peripheral equilibria (52–54). This may explain why none of these physiological changes is associated with thyroid dysfunction, although the equilibria between thyroid hormones and TSH are profoundly altered. Similar changes in control modes are observed in aging (55). In the Baltimore Longitudinal Study of Aging, some participants experienced a parallel rise/fall of TSH and FT4, whereas others showed a rising TSH with declining FT4 concentrations (55). Interestingly, the two patterns carried different mortality risks (55). Underlying mechanisms are complex and confounded by other pathologies, but pituitary responsiveness is clearly age related, as is deiodinase activity (56–58). Generally, higher TSH ranges are deemed acceptable in the elderly patient (57, 59, 60). More dramatic perturbations occur in severe non-thyroidal illness (NTI) including psychiatric disease (Figure 1C) where the allostatic stress response operates to alter both the setpoint and peripheral transfer parameters of the control loop, as reviewed elsewhere (36).

These lines of evidence suggest that equilibria and correlations between TSH, FT4, and FT3 are by no means invariant; rather being situationally adjusted in response to minor disturbances, and completely deranged in severe pathology (Figure 1B).

The pituitary–thyroid feedback loop must be revisited in the light of these findings (27). The strong log-linear correlation between TSH and FT4, observed in primary hypothyroidism, disintegrates toward the euthyroid range (61–65). The statistical correlation between TSH and FT4 is invalidated by clustering of genetically determined TSH–FT4 pairs (Figure 1B), and influences other than TSH shape the interrelations and equilibria (Figure 1C) (37, 65). This results in a high-individuality index of TSH, which gravely limits the use of population statistics in determining the euthyroid range (66, 67). Hypothalamic–pituitary–thyroid control is more complex and frequently denies the diagnostic simplification of a fixed log-linear TSH–FT4 gradient (Figures 1A–C) (27, 68). Studies in levothyroxine (LT4)-treated patients with thyroid carcinoma favor cascade-type control involving FT4, FT3 and their interaction over simple proportional control (38). Setpoint simulations corroborated these clinical results (37, 38). Together these findings question, the current diagnostic use of the TSH response as a reliable mirror of thyroid status. They demand a conceptional evolution of thyroid regulation to underpin a more differentiated clinical use.

## Appropriateness Versus Normality

The appropriateness of a TSH level is a different concept from range consideration. In secondary hypothyroidism or hypopituitarism, TSH measurement may be frequently inappropriate, residing within its reference range, yet abnormally low relative to the low-FT4 level. Deranged setpoints together with normal or elevated FT4 concentrations have been well recognized in TSH or thyroid hormone resistance syndromes and TSH producing pituitary tumors. Similarly, low-FT3 and/or low-FT4 serum concentrations in the NTI syndrome are accompanied by variable TSH values within or outside the reference range (4, 5, 36, 69–72). The isolated interpretation of TSH is not diagnostically useful in these conditions. While these limitations are well known, the dominance of TSH in the diagnosis of euthyroidism and as a treatment target for primary hypothyroidism also warrants closer scrutiny.

The high-individuality index of TSH and its correlative variation with thyroid hormones cause major statistical issues, because the basic concept of statistical hypothesis testing demands that the sample is randomly drawn from a population and thus representative of the population (73). This tenet is, however, violated where a measured TSH value is not an expression of normality surrounded by some margin of error or variation within a given population, rather a heterogeneous group of individuals sharing the same TSH value with different physiological meaning. For some individuals, the same TSH value may indicate a perfectly normal situation, for others subtle thyroid failure with declining FT4 concentrations and negative correlation between the two hormones, and for others a control shift to a positive TSH–FT4 correlation induced by non-thyroidal influences. A known response heterogeneity, however, precludes the use of statistical regression models based on normality assumption, requiring multilevel, hierarchical, or latent class models instead (74). The heterogeneity bias is known as Simpson’s amalgamation paradox (75, 76). Misleading and conflicting results may therefore be expected when mixing different underlying physiologies without proper stratification. Some authors reported a protective effect of TSH in the elderly, others an increased mortality risk of higher TSH within the normal range, and others no association of death with TSH, but with higher FT4 (21, 77–81). A dissociation between TSH and FT4 is also apparent in atrial fibrillation in euthyroid individuals where the risk increases with higher FT4 concentrations, but not lower TSH levels (82). We face a similar paradox in RCTs comparing T3–T4 combinations with T4 monotherapy, where patients invariably expressed their preference for the combination, but statistical analysis finds no evidence of superiority (83, 84).

We conclude that a TSH measurement representing ambiguous and overlapping categories between thyroid health and disease is by itself unreliable as a diagnostic tool. Statistical averaging or arbitrary cut-offs such as tertiles or quartiles should not be based on false assumptions of normality without further clinically meaningful stratification.

Another situation that differs from any naturally occurring condition is LT4 monotherapy for primary hypothyroidism. The homeostatic equilibria between TSH, FT4, and FT3 adequate for the previously healthy state no longer apply equally to the treated state, where dissociations between FT4 and FT3 and TSH and FT3 are apparent (Figures 1D,E) (31, 33, 65, 85–87). The T3/T4 conversion rate is reduced in LT4-treated athyreotic patients, compared with their rate prior to surgery (38, 86). FT3 is displaced from TSH in these patients, being relatively lower at the same TSH level (33, 85). While the consequences of the altered ratios are widely unknown and require further study (88), implications for the interpretation of TSH measurements can be readily derived.

Thyroid-stimulating hormone concentrations in normal health cannot be a therapeutic target for establishing LT4 dose adequacy (31, 89, 90). The treatment situation has to be re-evaluated on the basis of the shifted equilibria rather than pre-existing range considerations.

Can’t we just rely on bringing TSH within an acceptable population range and assume peripheral autoregulation at the tissue level should take care of the adequate tissue supply with T3? There are two major issues with this popular belief in the ability of the patient’s own pituitary gland—except for pituitary deficiency—to be the best judge of dose adequacy. First, this leaves patients frequently dissatisfied, because their quality of life is not generally restored with LT4 treatment to the same level seen in healthy persons despite their TSH concentrations being within the reference range (29). Second, the TSH for a patient on LT4 is not what it is for an untreated patient (65). The clinical and biochemical treatment response to LT4 turns out to be diverse and is influenced by many treatment-related or unrelated factors (Figures 1D,E) (38, 89). As a consequence of low-conversion efficiency, at least in some patients, the equilibrium for TSH may be shifted below the reference range of the healthy population (38, 86, 89). This poses a dilemma to clinicians.

## Toward an Optimized Treatment Strategy

Given the fundamental changes in thyroid control related to LT4 medication, we can no longer dismiss patient complaints when discrepancies arise between clinically hypothyroid versus biochemically euthyroid states (23–26). A recent retrospective study in patients with thyroid carcinoma followed long term found that displaced equilibria and resulting lower FT3 concentrations were associated with a lack of symptom relief, independently of known confounders such as gender, age, body weight, and weight-adjusted LT4 dose (38). This study and others contradict the assumption that patients may invariably gain satisfaction from LT4 treatment when TSH levels are kept within or even below reference range (29, 38, 90). Unfortunately, tissue T3 concentrations or T3 receptor saturation cannot be readily determined in humans. This was, however, done in rodents where various tissues remained in the hypothyroid state on LT4 monotherapy despite normal serum TSH levels, and only T3/T4 combination could restore euthyroidism in tissues (91, 92). These experiments uncovered limiting biochemical pathways that affect local sensitivity to T4 such as ubiquitination of hypothalamic type 2 deiodinase (92).

Implications emerging from advances in the understanding of the diverse facets of pituitary control (Figures 1A–E), clinical studies, mathematical simulations, and experimental treatments suggest replacing the current TSH paradigm with a more inclusive interpretation. This should take into account clinical signs and symptoms, all three thyroid parameters, and their relationships. The appropriateness of the respective levels relative to each other, to the previous healthy state and to a specific condition becomes more important than a blanket categorization according to placements within or outside any set range (Table 1).

**Table 1:** Differences between the thyroid-stimulating hormone (TSH) paradigm and the newly proposed paradigm.

TSH paradigm	New relational paradigm
Normality-based statistics	Homeostatic equilibria
Univariate normal distribution	Multivariate distributions
Population-based range	Setpoint, joined TSH–FT4 pairs
Low degree of individuality	High-individuality index
TSH is reflective of thyroid hormone status	TSH is interlocked with FT4 and FT3
The reference range is fixed across individuals and conditions	The setpoint is genetically determined and adjustable to various conditions
The parameters are treated as singularities, even when interpreted in combination	The parameters are interpreted in relation to each other
Interpreting ranges	Reconstructing setpoints
Levels are interpreted as within the reference range or outside its limits	Levels are interpreted as relatively appropriate or inappropriate
A TSH within its reference range in a healthy population indicates euthyroidism	The population-based TSH reference range is too wide to reliably define euthyroidism in a person
A high TSH indicates overt or subclinical hypothyroidism with rare exceptions	A high TSH originates from diverse physiologies
The setting of reference ranges and their interpretation is a simple process	The derivation of conjoined homeostatic equilibria is an intricate process
Subclinical thyroid disease entities are solely based on laboratory measurements and do not correspond to treatable clinical entities	The clinical change or challenge is considered primary mounting a defensive reaction that may alter the setpoint or transfer function
TSH is frequently interpreted without sufficient consideration of the clinical situation	The interpretation of TSH is tied to the clinical presentation
TSH reference range is universally suitable to judge treatment success	TSH level is inadequate as a measure of treatment success and LT4 dose adequacy
The suitable TSH range remains unchanged in LT4-treated patients	The suitable TSH range is shifted in LT4-treated patients
Exclusive role of TSH in guiding treatment targets	Supportive role of FT3 in guiding treatment targets

If required for symptom relief, considering wide variations in individual responses, clinicians may find it acceptable to suppress the TSH close to or slightly below its reference range while avoiding overt clinical or biochemical hyperthyroidism. In fact, in a recent study, patients after thyroidectomy with mildly suppressed TSH levels were closest to euthyroid, based on surrogate markers for end organ response, those with normal TSH mildly hypothyroid and those with strongly suppressed levels mildly hyperthyroid (90). T3/T4 combination therapy may be preferable to patients with persistent symptoms or a failure to sufficiently raise their FT3 concentration despite LT4 dose escalation and TSH suppression (93).

This practice should not be discouraged by incorrect statistical analyses of epidemiological studies. Many studies reported increased risks associated with suppressed TSH such as atrial fibrillation and osteoporosis but failed to properly classify the hormone status of patients into euthyroid versus hyperthyroid, and frequently did not even distinguish between treatment-induced TSH suppression and endogenous hyperthyroidism (94). Importantly, thyroid hormones, while suppressing pituitary TSH, have been reported to upregulate the locally produced osteoprotective TSHβv variant (95). Statistical associations with TSH cannot establish causality, as the opposing effects of low-TSH and low-FT3/TSHβv frequently occur together in LT4-treated patients.

However, it is equally appropriate to stress a caveat that not every patient on LT4 may require or tolerate a suppressed TSH. Unfortunately, conventional range considerations for TSH do not apply to the LT4-treated patient. There is no easy fix, but a paradigm shift could be a first step toward a solution. Until more personalized methods such as setpoint reconstruction have been evaluated (37), treatment adequacy must be judged on an individual basis by a combination of clinical and biochemical outcomes. The frequent overlapping and unspecific nature of hypothyroid symptoms presents yet another challenge (96–99). Unfortunately, reliable and readily accessible markers of the tissue effects are lacking.

However, the hypothyroid patient should not opt for under-treatment and forego symptom relief out of an exaggerated fear of over-treatment. On the other hand, long-term risks of LT4 treatment should also be carefully evaluated. Assessing scientific studies is a difficult task because statistical masking, confounding, and response heterogeneity can all be expected in a statistical parameter with a high-individuality index (67, 100, 101). This includes RCTs, regarded as the highest class of empirical evidence, which are not immune from violations of the underlying statistical assumptions.

Simplified concepts such as the negative thyroid pituitary feedback control contributed a fundamental understanding of endocrine principles, but were subsequently not refined for dealing with more complex facets of the various thyroid disorders and the clinical requirements of the diagnostic process. The pituitary TSH response is too diverse to be viewed as a sensitive mirror image of thyroid function. The interlocking elements of the control system are highly individual, dynamic, and adaptive. The assumed negative TSH–FT4 association that underpins the diagnostic use of TSH becomes frequently uncorrelated or even inverted, involving various influences, and mechanisms such as statistically multivariate clustering of TSH–FT4 setpoints, non-proportional cascade-type control, control mode shifts from negative feedback to positive tracking, and prevalent extra-pituitary–thyroid modulators of the relationship. Discrepancies arise between individually appropriate TSH levels and population-based reference ranges, laboratory-based disease definitions and treatment requirements, feedback adjustments and thyroid failure, and non-shared equilibria between thyroid health and LT4 treatment (Table 1). These have to be conceptionally reconciled to meet the needs of patients and clinical practitioners. TSH should be viewed as a controlling element and interpreted within its physiological role as a more narrowly defined and conditionally adaptive setpoint (Figure 1). Novel testable concepts are emerging and await clinical study.

## Author Contributions

RH and JM drafted the main part of the manuscript. RL and JD edited the text and contributed additional ideas, material, and text passages. JD also contributed to figures. All authors read and approved the final version.

## Conflict of Interest Statement

JD received funding and personal fees by Sanofi-Henning, Hexal AG, Bristol-Myers, Squibb, and Pfizer, and is co-owner of the intellectual property rights for the patent “System and Method for Deriving Parameters for Homeostatic Feedback Control of an Individual” (Singapore Institute for Clinical Sciences, Biomedical Sciences Institutes, Application Number 201208940-5, WIPO number WO/2014/088516). All other authors declare that there is no conflict of interest that could be perceived as prejudicing the impartiality of the research reported.
